# Fluorescent *Pseudomonas* -FAP2 and *Bacillus licheniformis* interact positively in biofilm mode enhancing plant growth and photosynthetic attributes

**DOI:** 10.1038/s41598-019-40864-4

**Published:** 2019-03-14

**Authors:** Firoz Ahmad Ansari, Iqbal Ahmad

**Affiliations:** Department of Agricultural Microbiology, Faculty of Agricultural Sciences, Aligarh Muslim University, Aligarh, 202002 India

## Abstract

Compatible interaction between commonly used plant growth promoting rhizobacteria (PGPR) in biofilm mode *in vitro* and in the rhizosphere is expected to provide better understanding for the development of effective consortium. With the above hypothesis, the present study evaluated two characterized PGPR (*Pseudomonas fluorescens* FAP2 and *Bacillus licheniformis* B642) for their biofilm-related functions using standard protocols. The interaction between the FAP2 and B642 in planktonic mode was studied by plate spot/overlay method and competitive growth assessment. Biofilm development on a microtitre plate and a glass surface was studied by standard methods. Biofilm formation was characterized by SEM. Rhizosphere and rhizoplane colonization of wheat seedlings by both isolates individually and by co-inoculation was studied by determining CFU/g of soil/root samples. Biofilm development on the root surface was further analyzed by SEM. Both isolates demonstrated multiple plant growth promoting (PGP) traits (production of IAA, siderophore, and ammonia; phosphate solubilization) and biofilm-related functions such as production of EPS, alginate, cell surface hydrophobicity and swarming motility. Both strains formed strong biofilms on a glass cover slip *in vitro*. Interaction between the two strains under the planktonic mode revealed no antagonism in terms of growth inhibition and competitive growth kinetics. Similarly, FAP2 and B642 strains formed a mixed biofilm on a glass cover slip as well as on seedling roots. Wheat rhizosphere and rhizoplane were colonized by both isolates as evidenced from their viable counts in single and co-inoculation. The effect of single and co inoculation revealed the significant enhancement of vegetative growth and photosynthetic parameters such as chlorophyll content, transpiration rate (E), internal CO_2_ concentration (Ci), stomatal conductance (g_s_), and net photosynthetic rate (P_N_) and leaf water potential (LWP) as compared to uninoculated control. Indigenous *Pseudomonas fluorescens* FAP2 strain and *Bacillus licheniformis* B642 are compatible PGPR in both planktonic and biofilm modes of growth and threfore could be developed effective consortium of PGPR. Further indepth investigation is required to understand molecular mechanism of the interaction in biofilm mode of growth under natural condition.

## Introduction

Soil microbial diversity and their functions are of prime importance to soil fertility. The rich diversity of soil microbe and microbial dynamics are maintained through multispecies interactions in both planktonic and biofilm modes^1^. It has been reported that environmental biofilms comprise the predominant mode of growth for bacteria, and soil surfaces provide for establishment of multispecies biofilms^2–4^. The biofilm mode of growth provides bacteria with protection against harsh environmental conditions and increased survivability by altering modes of growth and gene expression. Biofilms are also known to impart a several-fold increased tolerance to antibiotics, toxic chemicals and desiccation^5^. Different bacteria differ in their ability to develop biofilms *in vitro* and *in situ*^6,7^. Interaction between two organisms may be positive, negative, or neutral. It is believed that beneficial rhizobacteria, more accurately termed plant growth promoting rhizobacteria (PGPR), may increase their competitiveness and colonization through an enhanced capability of biofilm development^8^.

The performance of a microbial inoculant under field conditions is influenced by a suite of factors. The interaction between microbial inoculant in planktonic and biofilm modes may be positive for effective rhizosphere colonization and performance in the soil-plant system. The potential compatibility between Pseudomonas and Bacillus has been explored in planktonic and biofilm mode of growth. Both positive and negative interactions in mixed-species biofilms have been demonstrated^3^. Some workers have found that certain bacterial strains unable to form biofilms individually can promote a mixed-species biofilm, indicating a unique mode of cooperation in the biofilm mode^9–11^. Powers *et al*.^12^ demonstrated interspecies interaction between *Bacillus subtilis*, a common soil bacterium, and *Pseudomonas protegens*; metabolites produced by *P. protegens* inhibited biofilm formation and sporulation of *B. subtilis*^12^. It is not uncommon to determine antagonistic activity of one bacterium toward another in the planktonic mode of growth^5^. *Pseudomonas fluorescens* and *Bacillus* sp. are commonly used as bioinoculant for crop production and as biocontrol agents. However, to the best of our knowledge, no study has been conducted on their interaction in biofilm mode of growth. PGPR colonizes the roots of monocot and dicot and enhance the growth of plant by direct and indirect mechanisms. PGPR also modify the root functioning, improve plant nutrition and enhance physiological functions including photosynthetic parameters and water efficiency used in the leaf of plant^13^. It is hypothesized that promising PGPR exhibiting compatibility in planktonic and biofilm modes can deliver consistence performance in plant growth promotion. Therefore, our main objective in this study is to evaluate two promising PGPRs; *Pseudomonas fluorescens* FAP2 and *Bacillus licheniformis* B642 and their interaction in planktonic and biofilm modes. Further investigation relates to their interaction and significance in root and rhizosphere colonization, plant growth enhancement including some physiological attributes of photosynthesis.

## Results

### Characterization of plant growth promoting (PGP) activities, biofilm screening and identification of test bacteria

The FAP2 isolated after enrichment on King’s B medium was white with smooth edges and a convex surface. Additionally, the bacteria were motile, rod-shaped, and fluoresced yellow at 254 nm on the UV illuminator. The isolate reacted negatively to Gram-staining and showed positive results for citrate, catalase and oxidase, and reacted positively to the KOH solubility test (Table 1). To select indigenous fluorescent pseudomonas with multiple PGP activities, various strains were isolated and subjected to preliminary screening (data not shown). The FAP2 isolate demonstrated multifarious PGP activities; it produces indole acetic acid, siderophores, and HCN. Phosphate solubilizing and antifungal activity were also demonstrated. *Bacillus licheniformis* B642 exhibited similar PGP traits but did not showed antifungal activity, formed biofilm and exhibited biofilm-related functions (Table 1).

**Table 1:** Characteristics of *Pseudomonas fluorescens* FAP2 and *Bacillus licheniformis* B642 under study.

Characteristics	Pseudomonas fluorescens FAP2	*Bacillus licheniformis B642*
**Morphology and colony characteristics**	White in colour with smooth edge and convex surface.	Opaque with dull to rough surface, hair- like outgrowth attached strongly to agar,
		Whitish in colour.
**Biochemical test**	**Reaction**	**Reaction**
Gram reaction	−ve	+ve
Growth at 4 °C	+ve	−ve
Growth at 41 °C	−ve	+ve
Catalase	+ve	+ve
Oxidase	+ve	−ve
Starch hydrolysis	−ve	+ve
Gelatin liquefaction	+ve	+ve
Carbohydrate utilization		
Glucose	−ve	+ve
Sucrose	+ve	+ve
Mannitol	+ve	+ve
KOH solubility test	+ve	−ve
**PGP traits**	**Reaction (Value)**	**Reaction (Value)**
IAA	+ve (213.25 ± 1.32 µg ml^−1^)	+ve (190.23 ± 0.19 µg ml^−1^)
Phosphate solubilization	+ve (140.45 ± 1.32 µg ml^−1^)	+ve (110.12 ± 1.0 µg ml^−1^)
Siderophore	+ve (21.54 ± 1.06 µg ml^−1^)	+ve (30.85 ± 0.19 µg ml^−1^)
Ammonium	+ve	+ve
Hydrogen cyanide	+ve	−ve
Antifungal activity	+ve	−ve
**Salt tolerance (NaCl)**	800 m*M*	1 *M*
**MIC (mM)**		
**Biofilm associated traits**		
Swarming motility (diameter)	25 ± 1.56 mm	46 ± 2.12 mm
Swimming motility (diameter)	34 ± 2.02 mm	56 ± 2.08 mm
Cell surface hydrophobicity	68%	59%
EPSs production	+ve (1561.33 ± 1.05 µg ml^−1^)	+ve (234.16 ± 1.08 µg ml^−1^)
Alginate production	+ve (212.81 ± 1.06 µg ml^−1^)	ND
Biofilm formation (O.D)	Strong (1.12 ± 0.098)	Strong (0.95 ± 0.078)
**Molecular identification**		
16S rRNA partial gene sequencing	***Pseudomonas fluorescens FAP2***	***Bacillus licheniformis B642***

+ve stand for positive; −ve stand for negative reaction.

Identification of FAP2 was performed by partial 16S rRNA gene sequencing at DNA Sequencing Service (Macrogen Inc., Seoul, South Korea). The phylogenetic tree constructed from 16S rRNA gene sequence showed 99% similarity with *Pseudomonas fluorescens*.

### Quantitative estimation of PGP attributes

The isolate FAP2 showed a positive result for ammonia and hydrogen cyanide (HCN) production qualitatively, whereas B642 showed a positive result for ammonia and a negative result for HCN production. Antifungal activity of FAP2 was demonstrated against *Alternaria solani, Aspergillus niger and Fusarium oxysporum* whereas B642 revealed no antifungal activity. Biochemical and plant growth promoting activities of both test bacteria are depicted in Table 1. Quantitative estimation of IAA in the presence of an exogenous supply (500 µg ml^−1^) of tryptophan revealed 213.25 ± 1.32 µg ml^−1^ and 190.23 ± 1.19 µg ml^−1^ production in FAP2 and B642, respectively. Similarly, solubilization of tricalcium phosphate was 40.45 ± 1.32 µg ml^−1^ for FAP2 and 110.12 ± 1.0 µg ml^−1^ for B642.

### Alginate and EPS quantification

Alginate production was found 212.81 ± 1.06 µg ml^−1^ by FAP2 while B642 could not produce alginate. Additionally, exopolysaccharides (EPS) measured 1561.33 ± 1.05 µg ml^−1^ in FAP2 whereas B642 produced 234.16 ± 1.08 µg ml^−1^ EPS (Table 1).

### Cell surface hydrophobicity

The isolate FAP2 revealed more hydrophobicity (68%) to hydrocarbons as compared to B642 (59%) (Table 1). When both strains were mixed in a 1:1 ratio, a slightly increased hydrophobicity (76%) was recorded.

### Swimming and swarming activity

The bacterial swimming and swarming motility was demonstrated quantitatively. After 48 h incubation, FAP2 showed little swarming motility with swarm diameter upto 25 ± 2.02. Swimming motility was expressed up to 34 ± 1.56 mm. On the other hand, B642 exhibited greater motility as compared with 56 ± 2.08 mm diameter for swimming and 46 ± 2.12 mm for swarming **(**Table 1).

### *In vitro* interaction between FAP2 and B642

**Figure 1:**
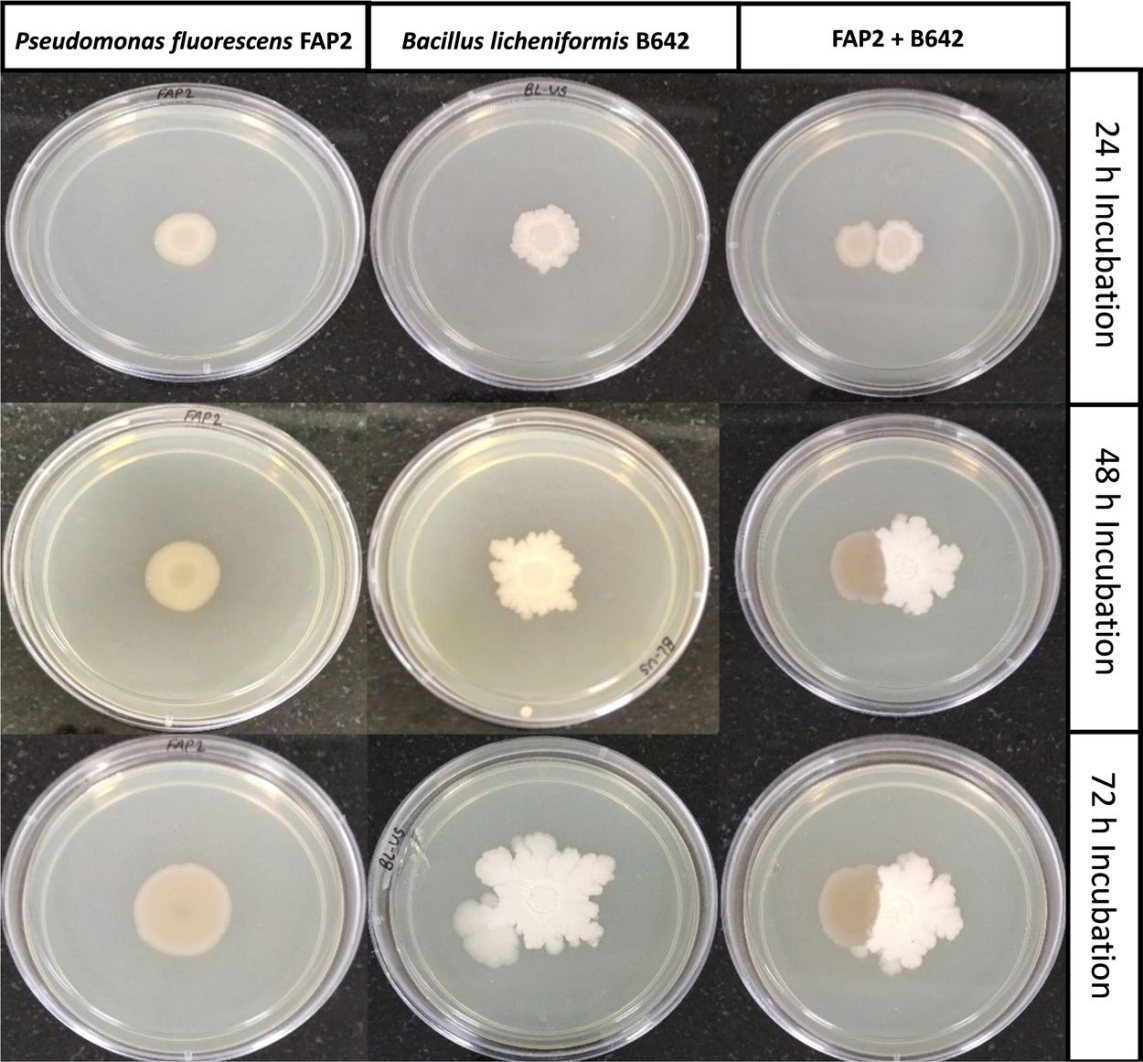
Positive interaction between *Pseudomonas fluorescens* FAP2 and *Bacillus licheniformis* B642 on the solid agar media at different time intervals using co-cultured plate method. The centers of the initial cell suspensions were placed 0.5 cm away from each other.

**Figure 2:**
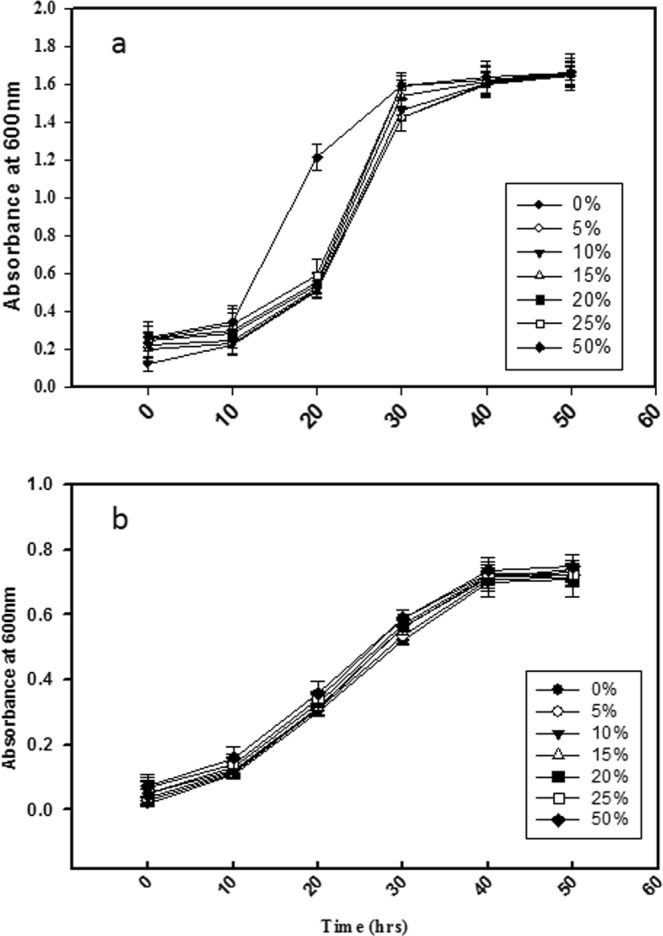
Competitive growth assessment of Pseudomonas fluorescens FAP2 and Bacillus licheniformis B642. (**a**) Filtered supernatant of FAP2 supplemented at 0% to 50% in NB medium to check the growth of B642 at different time interval (**b**) Filtered supernatant of B642 supplemented at 0% to 50% in NB medium to check the growth of FAP2 at different time interval.

Early in the study a compatibility assay was performed among 10 potential PGP strains using a co-cultured plate, overlay and well diffusion method (data not shown). Based on preliminary compatibility assay, two strains, i.e., FAP2 and B642 were found to be compatible. *In vitro* interaction between the above two strains was examined on the solid agar media which was co-cultured for 24 to 72 h. Both strains interacted and no antagonistic relationship was found **(**Fig. 1). We also observed that there was no antagonistic interaction between FAP2 and B642 by overlay method. Growth patterns were observed for FAP2 and B642 to determine the effect of different concentrations of 48-h culture supernatant compatibility among bacteria. The absorbance at 600 nm at different time intervals (0–50 h) and viable counts (CFU/ml) were not found significantly different with control (Fig. 2).

### Biofilm development on microtitre plate

An assay for biofilm formation was performed on microtitre plates using crystal violet. The isolate FAP2 showed strong biofilm development with optical density 1.12 ± 0.098 at 590 nm. The B642 was assessed as a strong biofilm former with optical density (0.95 ± 0.078), which was comparatively low to that of FAP2 (Table 1).

### Single and mixed biofilm development on glass surface

**Figure 3:**
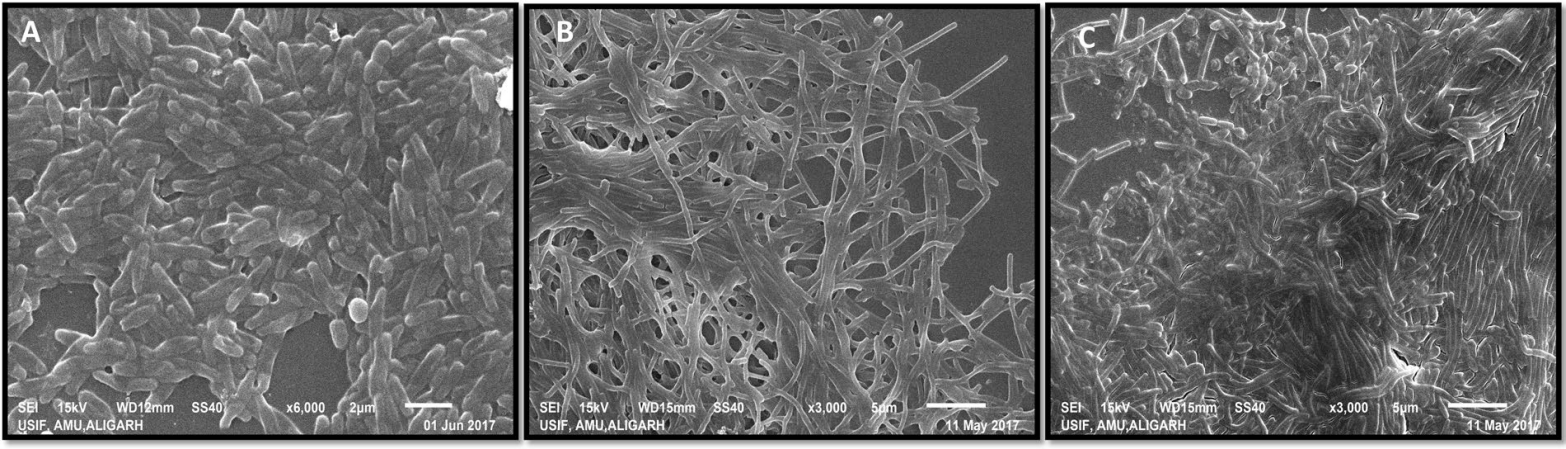
Scanning electron micrograph of biofilm formation on glass surface by *Pseudomonas fluorescens* FAP2 (**A**) and *Bacillus licheniformis* B642 (**B**) individually and in consortium (**C**).

An assay for single and mixed biofilm development on the glass coverslip surface was performed in a 12- well tissue culture plate. Both test bacteria (FAP2 and B642) formed strong biofilms individually (Fig. 3A,B) as well as in mixed biofilms (Fig. 3C).

### Single and mixed biofilm formation on root surface

**Figure 4:**
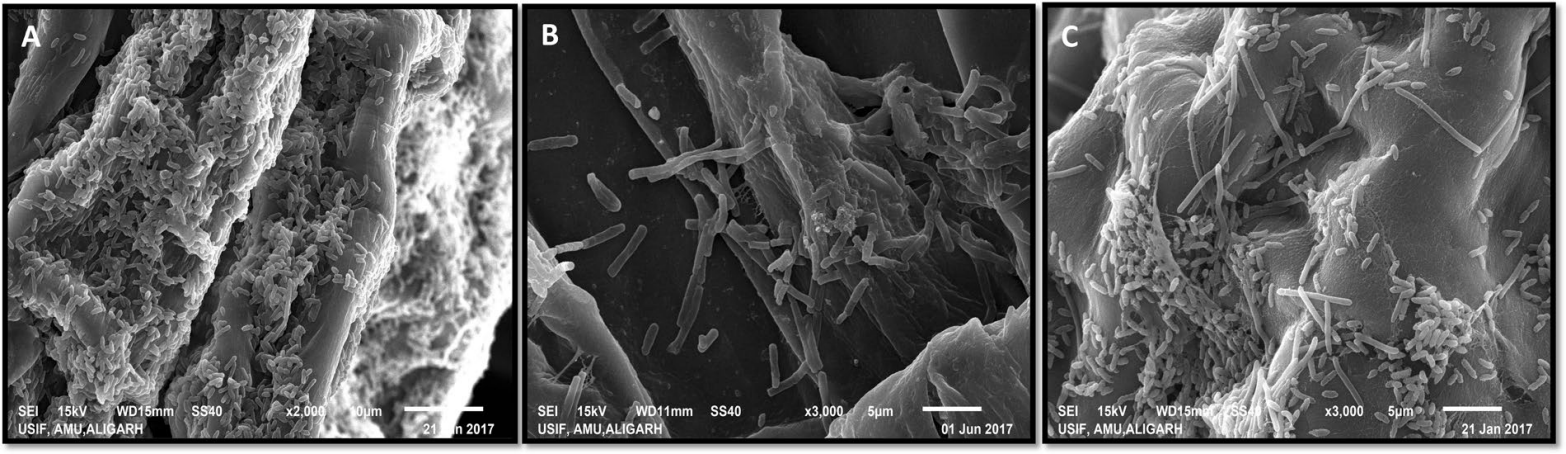
Scanning electron micrograph of *Triticum aestivum* seedling roots colonized by *Pseudomonas fluorescens* FAP2 (**A**) and *Bacillus licheniformis* B642 (**B**) individually and in consortium (**C**).

Adherence of bacteria to the root surface of wheat (*Triticum aestivum*) was visualized after incubating 7-day-old surface sterilized seedlings with the freshly grown culture of FAP2 and B642 individually as well as in mixed (1:1) form. Both strains adhered to the root surfaces *in vitro* alone. The strain FAP2 and B642 attached to the root surface almost at an equal density at a different portion of the root. Similarly, Scanning Electron Microscopy (SEM) images of root tips dipped in the culture of above strains individually (Fig. 4A,B) and in the mixed formulation (Fig. 4C) also showed strong adherence by both bacteria simultaneously. It was also evident that both strains have an equal affinity to adhere the root surface without affecting each other, thus further confirming their compatibility.

### Rhizoplane and rhizosphere colonization

**Figure 5:**
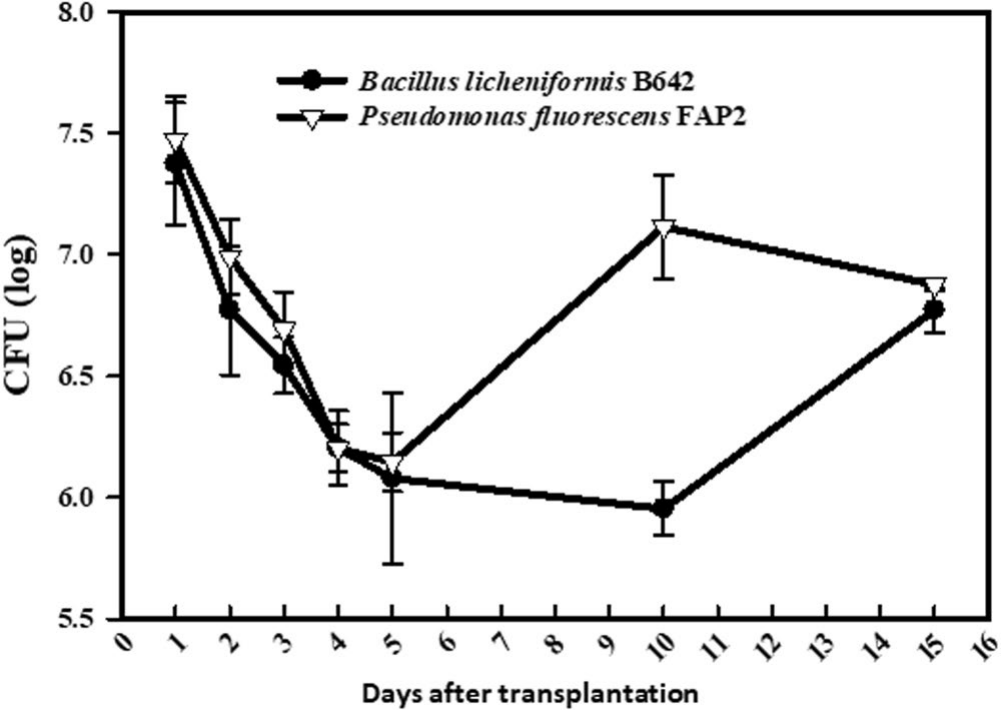
Rhizoplane colonization by consortium of FAP2 and B642 under microcosm soil system at different days after transplantation.

**Figure 6:**
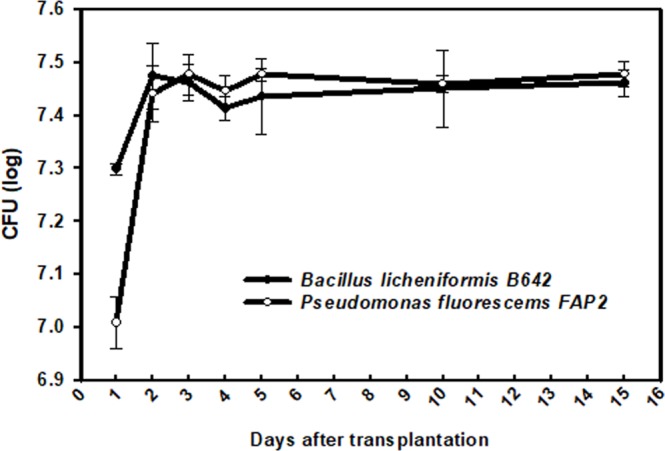
Rhizosphere colonization by mixed culture of FAP2 and B642 under microcosm soil system at different days after transplantation.

Rhizoplane and rhizosphere colonization was studied separately on treated seedlings after transplantation. At 24 and 48 h after transplantation of treated seedlings, the structure of the biofilm on the root surface was drastically altered and relocated from the rhizoplane to rhizosphere. Initial counts of FAP2 was 7.4 log CFU gm^−1^ of root; when transplanted into the sterile soil system the biofilm on the wheat root surface declined in the rhizoplane from 7.4 log CFU to 5.9 log CFU gm^−1^ of root. Rhizoplane colonization of B642 also diminished at one and two days after transplantation. Values as the viable count of the rhizoplane declined from 7.3 log CFU to 5.6 log CFU gm^−1^ of root (Fig. 5). We carried the experiment for up to 15 days after transplantation; after three days of transplantation, the biofilm on the rhizoplane was declined significantly (Fig. 5). In the case of FAP2, after 5 days since transplantation there was a reverse back of FAP2 population to the rhizoplane from the rhizosphere. Similar behavior of colonization was also evident by B642 strain. After 10 days of transplantation, B642, demonstrated recolonization of root surface probably by movement from the rhizosphere to rhizoplane. Both strains coexisted and showed compatibility in significant numbers in the rhizosphere up to 15 days since transplantation (Fig. 6).

### Soil Plant Analysis Development (SPAD) chlorophyll, photosynthetic attributes and Water Use Efficiency (WUE)

**Figure 7:**
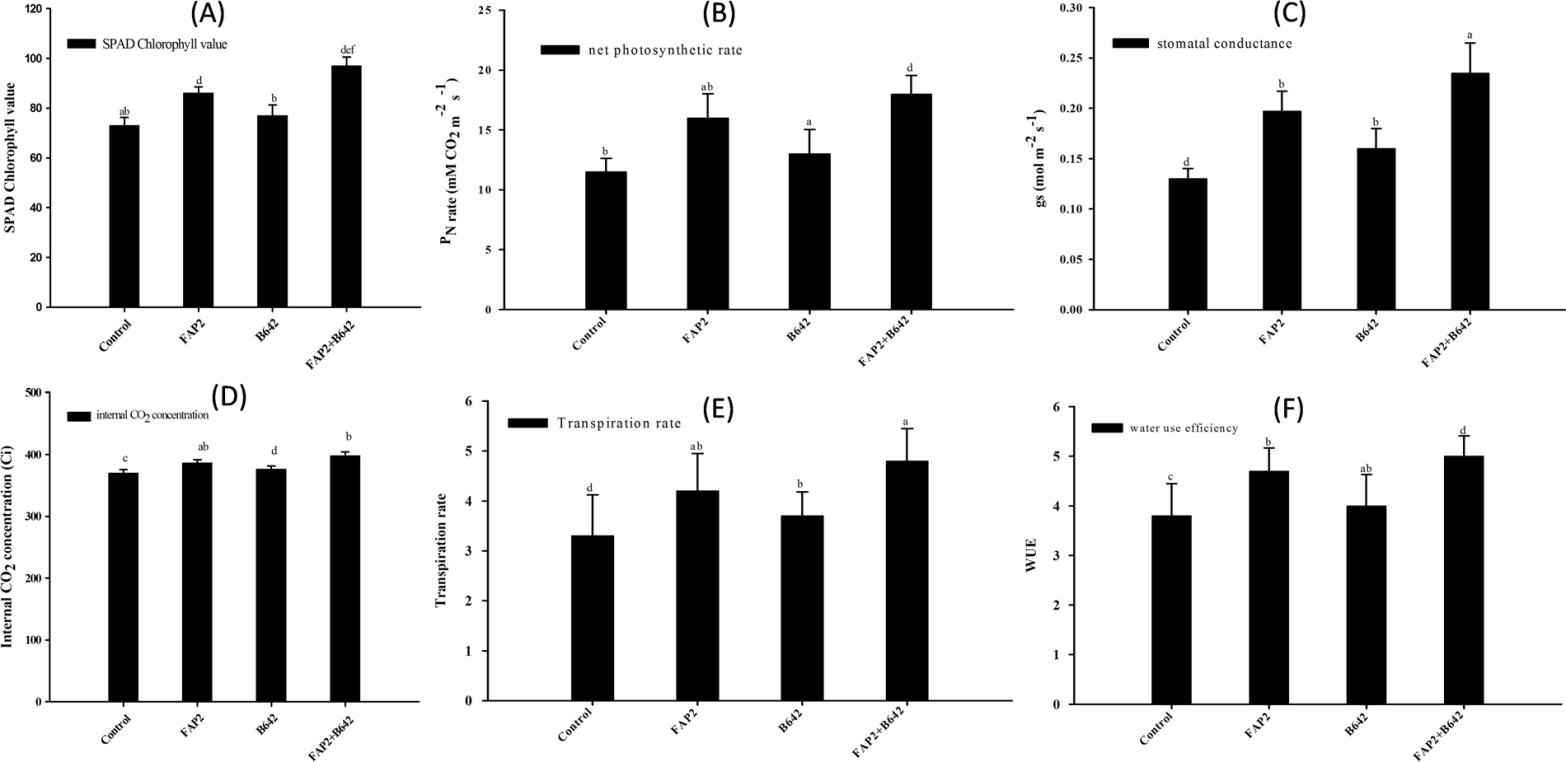
Effect of plant growth promoting rhizobacteria FAP2 and B642 individually and in consortium on the attributes of photosynthesis (**A**) SPAD chlorophyll value, (**B**) net photosynthetic rate [P_N_], (**C**) stomatal conductance [g_s_], (**D**) internal CO_2_ concentration [C_i_], (**E**) transpiration rate [E] and (**F**) water use efficiency [WUE].

All the physiological traits of the treated plants were analysed at 30 day after seeding. The SPAD chlorophyll content was measured with single and consortium of tested strains. The chlorophyll content in fresh leaf was enhanced 22.9% by FAP2 and 11.4% by B642 in single inoculation. Interestingly, the content of chlorophyll was increased by 34.3% significantly when plants were inoculated with consortium of both strains (Fig. 7A). The net photosynthesis rate was increased by 45.5% with the inoculation of FAP2 and 18.2% by B642. Attractively, plants inoculated with consortium of FAP2 and B642, the rate of photosynthesis was raised by 63.6% significantly compared to uninoculated control (Fig. 7B). Moreover, the stomatal conductance was increased by 53.8% in FAP2 inoculated plants and B642 increases stomatal conductance (23.1%) alone. Interestingly, on the application of consortium of FAP2 and B642, stomatal conductance was enhanced by 69.6% significantly compared to uninoculated control (Fig. 7C). The internal CO_2_ concentration in the inoculated plants by FAP2 and B642 was raised by 8.6% and 2.9% respectively alone but in the presence of consortium of both strains, the concentration of internal CO_2_ was enhanced significantly by 14.3% over uninoculated control (Fig. 7D). At the same time, transpiration rate was also monitored which was increased 27.3% and 18.2% in FAP2 and B642 inoculated plants respectively. Attractively, 45.5% transpiration rate was raised significantly on the inoculation of consortium of the both strains (Fig. 7E). Leaf water potential was also enhanced on the inoculation of FAP2 and B642 by 23.7 and 7.9% individually and 36.8% by the application of consortium compared to control (Fig. 7F).

### Measurement of plant growth attributes

**Figure 8:**
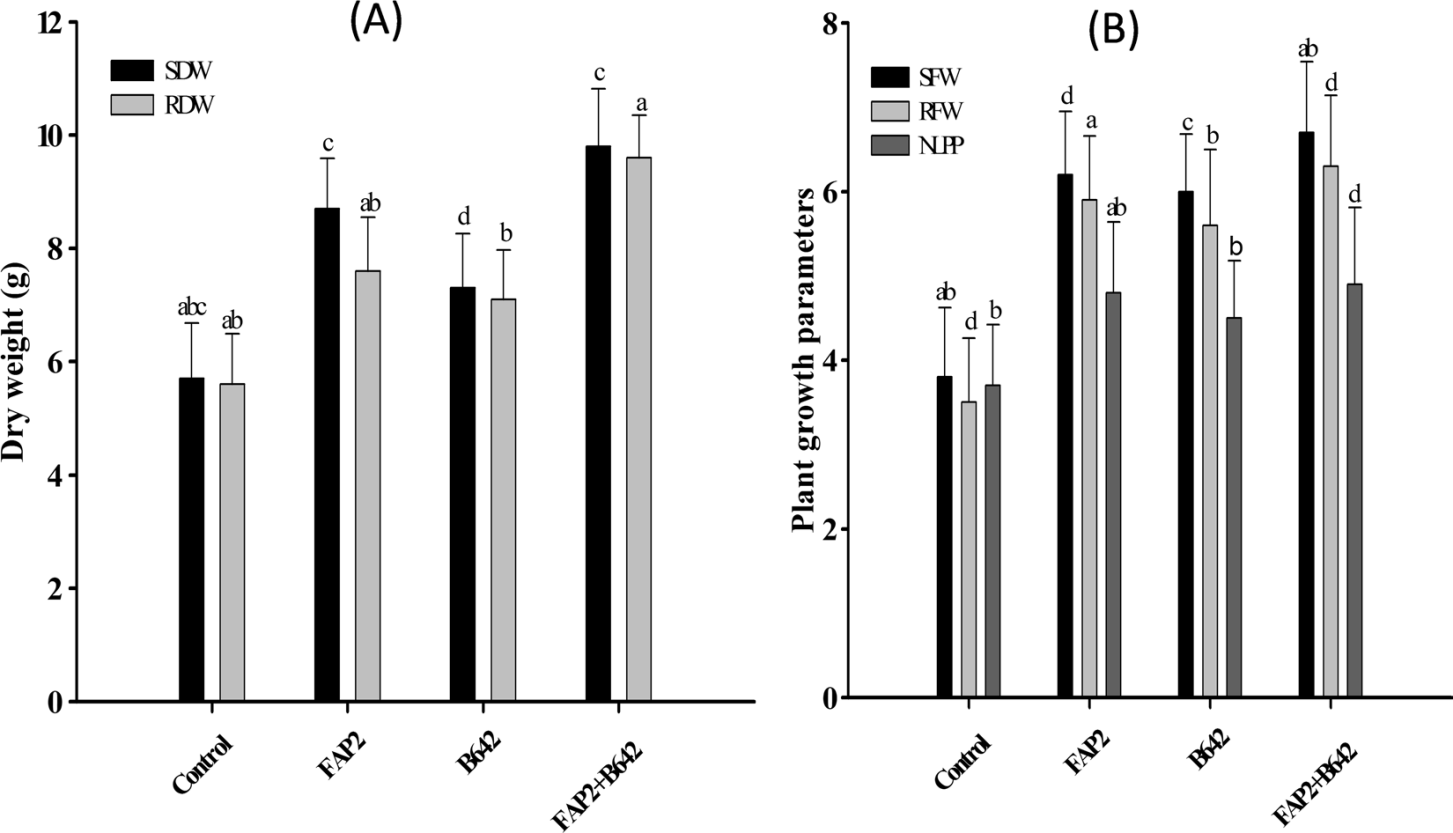
Effect of FAP2 and B642 alone and in consortium on plant growth attributes, (**A**) shoot dry weight [SDW] and root dry weight [RDW]; (**B**) shoot fresh weight [SFW], root fresh weight [RFW] and number of leaf per plant [NLPP].

Plant growth parameters such as shoot fresh weight, root fresh weight, shoot dry weight; root dry weight and number of leaf per plant were assessed after 90 days of seeding. Briefly, shoot fresh weight was enhanced 50% and 13.2% by FAP2 and B642 applied alone. Interestingly, on the inoculation of consortium of both strains shoot fresh weight was raised by 97.4% significantly over un-inoculated control. Similarly, the root fresh weight was also increased 39.4% by FAP2 and 48.5% by B642 inoculation. Interestingly, there was 81.8% root fresh weight increased on the inoculation of consortium. Similar pattern was observed in shoot and root dry weight by individually and consortial effect of tested strains. Shoot dry weight was enhanced 59.3%, 31.5% by FAP2 and B642 respectively, and 79.6% enhancement was recorded on the consortial application. Root dry weight was raised 46.2% and 21.2% on the inoculation of FAP2 and B642 individually. Similarly, root dry mass was enhanced by 76.9% by the consortium application (Fig. 8A,B). Number of leaf per plant was also vary and significantly enhanced on the inoculation of individually and consortia as depicted in Fig. 8.

## Discussion

The use of bioinoculants for promoting plant growth and protecting plant health is an integral component of sustainable crop production. Efficacy of bacterial inoculants on crop production depends upon complex processes of plant-microbe interactions and environmental factors^14^. Successful rhizosphere colonization by bacterial inoculants and the compatible nature of co-inoculants are key factors in consistence performance of bioinoculant^15–17^. However, the intrinsic ability of bacteria to adhere and form biofilm *in vitro* and in association with plant roots may vary^6^. It has been demonstrated that interactions of bacterial populations and with plants occur predominantly in biofilm mode rather than planktonic mode, and these interactions may be positive or negative^3,18,19^. Therefore establishing a positive interaction among bacterial inoculants both in planktonic and biofilm mode is a prerequisite for successful establishment of the inoculants in the rhizosphere. In this study, we found that two PGPR (FAP2 and B642) are compatible in the *in vitro* under planktonic mode of growth assessed by competitive growth assessment between the two PGPR strains in the presence of their extracellular metabolic products as well as cell to cell contact basis on agar plate also revealed similar results. Such interactions between different PGPRs have been reported earlier^19^. However, negative interactions among rhizobacteria such as *Bacillus subtilis* and *Pseudomonas protegens* were also documented^12^. The interactions among rhizobacteria are dependent upon the nature of the strains under study, and their metabolic requirements and extracellular products.

Based on *in vitro* compatibility in the planktonic mode, we further investigated biofilm formation on a glass surface in a 12-well tissue culture plate. Both the PGPR strains (FAP2 and B642) formed strong biofilms individually and also in mixed cultures. This provides evidence for a compatible nature of both PGPR in biofilm mode of growth. Such compatibility in mixed biofilm formation among different soil bacterial isolates was demonstrated previously^19^. We further investigated the interaction between FAP2 and B642 on wheat seedling root surfaces regarding adherence and development of microcolonies/biofilms. Root SEM analysis revealed that both strains adhered to root surfaces and formed biofilms individually. FAP2 formed more dense biofilm compared to B642; however, in mixed treatment both cultures occupied root surfaces spatially separated as well as in mixed form. These observations clearly demonstrated that two inoculants are also compatible in association with plant root surface biofilm development. Our finding of mixed root surface biofilm formation by these strains is probably the first report in this direction. In a separate experiment, we transplanted bioinoculant treated wheat seedlings by both cultures individually and in mixed form in sterile soil microcosms. Viable counts of two inoculants in the rhizosphere and rhizoplane of the wheat plant was examined daily from day 1st to 5th and then after 10th and 15th days following transplantation. Rhizosphere and rhizoplane viable counts at different times varied, indicating that after transplanting seedlings in soil the adhered bacterial cultures relocate and become established in both rhizosphere and rhizoplane zones. The findings indicated that inoculated cultures survive successfully in compatible manner but under goes adjustment in the distribution pattern in rhizosphere and rhizoplane zone. Interaction of two bioinoculants in mixed biofilms *in vitro* has been recently demonstrated between *Stenotrophomonas*, *Xanthomonas* and *Microbacterium* and *Paenibacillus*^3^. However, colonization of root surface and rhizosphere in mixed biofilm is poorly explored. Further, monitoring bioinoculant in the rhizosphere and on root surface under natural condition is challenging. The use of molecular techniques might be helpful in exploring such interaction *in situ* as the nature of interaction will be multispecies. Inoculation with PGPR (Pseudomonas and Bacillus) to wheat has been documented on plant growth, yield and physiological attributes by several workers^20,21^. However, in this study FAP2 and B642 both individually and in combination influence significantly the plant growth parameters analyzed at 90 days of inoculation. Similarly, photosynthetic attributes estimated a 30 day of inoculation showed a positive indication for enhancement of plant growth promotion compared to control. The above data are supportive that both strain (FAP2 and B642) survived in association with root zone and enhances plant growth significantly which are in agreement with available reports on wheat with other PGPR^22^.

## Conclusions

The present study highlights compatible nature of FAP2 and B642 and provides the first insight in interaction studies in the planktonic and biofilm mode of growth and its relevance in rhizosphere and root surface colonization, plant growth stimulation and physiological attributes modulation. The significance of biofilm development in these interactions may be extended to other PGPRs to evaluate its significance in soil-plant system under multispecies interactions.

## Materials and Methods

### Bacterial strains and their characteristics

Rhizospheric soil samples of wheat (*Triticum aestivum*) were collected aseptically and processed for enrichment before isolating fluorescent Pseudomonas on Kings B agar medium (Hi-media) containing the following per liter of distilled water: 10 g proteose peptone, 10 ml glycerol, 1.5 g K_2_HPO_4_, 1.5 g MgSO_4_ and 20 g agar, adjusted to pH 7.2. *Pseudomonas fluorescens* was detected under a UV illuminator at 254 nm and colonies selected and purified. The suspect culture was examined for cultural and biochemical characteristics before assigning the isolate number for preservation and further identification. Promising PGP Pseudomonas isolates were selected for the laboratory culture collection. One isolate was identified as *Pseudomonas fluorescens* FAP2 by 16S rRNA partial gene sequence analysis with assigned accession number (KY110950) was included in this study.

*Pseudomonas fluorescens* FAP2 was isolated from rhizospheric soil and selected after a screening of isolates using standard protocols described elsewhere^23^. *Bacillus licheniformis* B642 was obtained from the NRRL (USDA) culture collection. Both cultures were subjected to morphological and biochemical tests including such as catalase, oxidase, starch hydrolysis, gelatin liquefaction and carbohydrate utilization as per Bergey’s Manual of Systematic Bacteriology. All experiments were conducted with three replications and repeated at least two times.

### *In vitro* assessment of plant growth promoting attributes of test isolates

Qualitative estimation of the production of indole acetic acid (IAA) was detected by the method as mentioned earlier^24^. The method of Loper and Scroth (1986) for IAA production in the presence of 500 μg ml^−1^ tryptophan as adopted by Ahmad *et al*.^25^ was used for quantitative estimation of IAA^25,26^. Ammonia production in peptone water was carried out using the method of Dye^27^. Production of hydrogen cyanide (HCN) among test strains was screened using the method of Lorck^28^. Quantitative estimation of siderophores was by the method described earlier^29^. Qualitative estimation of phosphate solubilization was carried out by observing a halo zone around a bacterial colony growing in Pikovskaya medium^30^. Solubilization of tricalcium phosphate in liquid medium was performed as described earlier^25^. Antifungal activity was determined by observing clear zones around bacterial colonies growing in potato dextrose agar medium (Hi-media)^31^.

### Quantitative estimation of exopolysaccharides (EPS) extraction

EPS extraction and quantitation was performed as described earlier^32^. The pre-inoculum was grown in KB broth (Hi-media) for FAP2 and Nutrient Broth (NB) for B642 overnight at 28 ± 2 °C. A volume of 500 µl of pre-inoculum was added to fresh 50 ml culture medium and allowed to grow at 28 ± 2 °C for five days at 120 rpm in a rotatory shaking incubator. The culture volume of 200 ml was centrifuged at 11500 rpm for 20 min at 4 °C. The supernatant was filtered through a nitrocellulose filter with a pore size of 0.45 µm (Millipore filter, Bangalore India). From the final filtrate, EPSs were precipitated after addition of three volumes of chilled ethanol. The solution was stored at 4 °C overnight for precipitation of exopolysaccharides. The weight of the precipitated EPS was measured after drying at 80 °C for 48 h.

### Alginate quantification assay

Alginate extraction from bacterial strains was performed with the 48-h culture. After incubation, the culture was centrifuged at 10,000 rpm for 10 min and the cell-free supernatant was collected. Isolation of deacetylated alginate from culture supernatants was performed by adding an equal volume of isopropanol and storing for one day under static conditions. The precipitate was collected by centrifugation 10,000 rpm for 10 min. The resultant pellet was washed successively with 1 ml of 70% and 96% ethanol. The pellet was dried at 37 °C for 15 min and dissolved in 1 ml of sterile double distilled (dd) H_2_O. For quantification, 100 μl of the suspension was transferred to fresh test tubes and volume made up to 1 ml with Milli-Q water. One ml of freshly prepared borate sulfuric acid solution was added followed by 30 μl of fresh carbazole reagent and mixed thoroughly. The mixture was kept for 15 min at room temperature and absorbance measured at 500 nm against a reagent blank. Alginate quantity was calculated in terms of μg/mg wet biomass^33^. Every assay was repeated in triplicate at least three times.

### Cell surface hydrophobicity assay

Cell surface hydrophobicity (CSH) was quantified using a microbial adhesion assay to hydrocarbons (MATH) as described earlier^34^. Cultures of FAP2 and B642 were incubated in NB broth and hydrophobicity was determined after the first, second and fourth days of incubation. To evaluate percentage hydrophobicity, 5 ml of 24 h-grown culture was centrifuged at 8000 rpm for 10 min and pellets were resuspended in phosphate-magnesium buffer (pH = 7.4). Absorbance was read at 400 nm using a UV-vis spectrophotometer [Shimadzu UV-vis spectrophotometer-8500 II] and designated the initial bacterial suspension. Five ml of culture was added to 0.2 ml of hexadecane and mixed vigorously with a cyclomixer (REMI, CM-101PLUS). After separating the aqueous phase, absorbance was determined at 400 nm and designated the final concentration in the aqueous phase. Percentage of hydrophobicity was calculated as follows:

$${\rm{Percent}}\,{\rm{hydrophobicity}}( \% )=[{\rm{1}}\,-\,({{\rm{A}}}_{1}/{{\rm{A}}}_{{\rm{0}}})]\ast {\rm{100}}$$

where A_1_ is initial bacterial suspension absorbance and A_0_ is absorbance of the aqueous phase.

### Swimming and swarming motility assay

Swimming and swarming motility was performed according to the method adopted previously^35^. Assay plates containing nutrient broth medium with 0.3% and 0.5% (w/v) agar were used for swimming and swarming motility assays, respectively. Each assay plate was spot inoculated with 3 µl of freshly grown bacterial culture (cell density 10^7^ cells ml^−1^). Inoculated plates were sealed with parafilm to prevent dehydration and incubated at 28 ± 2 °C for 48 h. Swimming and swarming motilities were subsequently determined by measuring the swarm diameter after 24 and 48 h and expressed in mm.

### Interaction between bacterial cultures under planktonic mode of growth

The interaction between bacteria was studied by co-culture plating and by the overlay method in the same culture medium, followed by observation of growth inhibition^36^. Co-inoculation of freshly grown culture in a 1:1 ratio in fresh medium was also assessed by CFU count and O.D. determination for individual and mixed cultures.

Determination of bacterial compatibility was also tested by overlay method using soft agar. In this test, the bacterium was mixed homogenously in moderately cooled soft agar and gently poured over the surface of primary layer previously inoculated with other test isolate^37^. Further, plates were incubated for 48 h at 28 ± 2 °C and following the incubation, a measurement in the term of growth inhibition among these tested isolates (FAP2 and B642) was executed to assess their compatibility.

### Effect of supernatant of bacteria over planktonic growth of other bacteria

Cultures of FAP2 and B642 were grown separately in NB for 48 h at 28 ± 2 °C in a rotary shaker incubator at 180 rpm. After incubation, cultures were filtered using 0.22 µm filter paper (PALL Corporation, USA). The sterile culture filtrate was mixed at concentrations of 5, 10, 15, 20, 25 and 50% (v/v) in the fresh NB medium. The growth of one bacterial isolate in the filtered supernatant of the other bacterial culture was plotted at different time intervals, e.g., 4, 8, 24, 36 and 48 h by reading absorbance at 600 nm using a UV-visible spectrophotometer [Shimadzu UV-vis spectrophotometer-8500 II]^38^.

### Biofilm quantification using crystal violet assay

*In vitro* biofilm quantification was performed using the method mentioned previously^39^. Bacteria grown overnight on a NA plate was resuspended in NB medium and diluted to the final optical density at 600 nm of 0.02. Bacterial cultures were transferred to a 96-well polystyrene plate. The volume of culture was 160 µl per well. Cultures were allowed to stand at 28 ± 2 °C for 48 h. Quantification of biofilm formation was assayed by staining with 0.1% crystal violet. After the growth period, wells of the microtitre plate were emptied and washed gently with dd H_2_O at least three times to remove loosely attached bacterial cells and left at room temperature for 30 min. Samples were stained by addition of 200 µl of 0.1% crystal violet solution to each well and incubated at 28 °C for 20 min. The wells of plates were then washed. The intensity of crystal violet staining was measured after addition of 70% ethanol to each dry well. Samples were incubated for 20 min after which the absorbance was measured at 590 nm on an ELISA plate reader (Thermo Scientific Multiskan EX, UK). All samples were tested in five replicates.

For mixed biofilm development, an 80 µl culture of each species was added in a 1:1 ratio in each microtitre well. After 24 h incubation, biofilm formation was quantified using a modified crystal violet assay^38^. The mixed species containing FAP2 and B642 strains was examined for synergy. The inoculum value of each bacterium was equivalent and added to make a total of 160 µl. The biofilm experiment was repeated at least thrice over three independent days with five to seven replicates.

### Biofilm formation ***in vitro*** and visualization by SEM

Freshly grown bacterial culture (2 ml culture and 2 ml broth) were kept in a 12-well tissue culture plate (Hi-media). A glass cover slip (20 mm) was placed in each well and incubated at 28 ± 2 °C for 24 to 48 h under static conditions. After the incubation period, glass cover slips were washed three times with 1x phosphate buffer saline (pH 7.2) to remove loosely attached cells. The biofilm on the glass surface was fixed using 2.5% glutaraldehyde (v/v) in 0.1 *M* phosphate buffer (pH 7.2) for 4 h. Samples were dehydrated through a graded ethanol series 30–100% (v/v) and the samples were dried using critical point dried in CO_2_ and shadowed with gold for viewing under a scanning electron microscope (Model: JOEL, 76510LV, Japan) at University Sophisticated Instrumentation Facility, AMU, Aligarh. For mixed biofilm development on the glass surface, the freshly grown bacterial culture was mixed in a 1:1 ratio and then prepared for scanning electron microscopy as described above.

### Rhizoplane and rhizosphere colonization

The bacterial colonization of the rhizosphere and rhizoplane was demonstrated quantitatively. The method of the rhizoplane and rhizosphere colonization was adapted as described previously with minor modifications^40^. Briefly, sandy clay loam soil (250 g) in an autoclavable plastic pot was maintained in a climate-controlled growth chamber. Plant nutrient solution (10% v/w) was applied to moisten the soil. For the root colonization experiment, seven day-old aseptically grown wheat seedlings at the two to three-leaf stage were dipped into overnight-grown test cultures separate it and in mixed (1:1) ratio for 4 h. The treated seedlings were transplanted to a sterile soil microcosm in small plastic pots and kept in the climate-controlled growth chamber. At daily intervals (1–15 days after transplantation), seedlings were uprooted and washed gently in sterile distilled H_2_O to remove loosely attached soils from the root surface. Further, roots (1 g) were removed from seedlings and macerated aseptically using a mortar and pestle and appropriately diluted in sterile Normal saline solution (NSS) to study rhizoplane count. The CFU were determined by plating 100 µl of the sample on nutrient agar plates amended with 30 µg/ml of rifampicin for selective isolation and incubated at 28 ± 2 °C for 48 h^41^. Furthermore, at the same time intervals, (1, 2, 3, 4, 5, 6, 7, 8, 9, 10 and 15 days), rhizospheric soil, was collected and serially diluted into the NSS. A volume of 100 µl of each dilution was plated onto nutrient agar medium containing rifampicin. Plates were incubated at 28 ± 2 °C for 48 h and CFU/gm of rhizospheric soil was counted.

### Biofilm development on root surface

Another experiment assessed biofilm development by the two strains. Healthy and uniform size seeds of wheat (*Triticum aestivum)* (cv. 343) were surface sterilized with 70% ethanol for 3 min. followed by 4% sodium hypochlorite, 3 min, washed five times with changes of sterile water and dried under shade. Surface sterilized seeds were then sown in the soil microcosm. After seven days of sowing at two to three leaf stage the wheat plants uprooted from soil and washed two to five times to remove all the soil particle form the root surface. The roots of the wheat plant were dipped in overnight-grown culture and transplanted in the gnotobiotic soil system. Subsequently, the plants were uprooted at different time intervals and washed to remove loosely attached cells from root surface. Root samples were then fixed in 2.5% glutaraldehyde (v/v) in 0.1 M phosphate buffer (pH 7.2) for 4 h^42^. The root samples were dehydrated through a graded ethanol series (30–100%, v/v) and critical point dried in CO_2_. To prevent tissue damage, pressure was released slowly and shadowed with gold (22 nm) before viewing under a scanning electron microscope (JOEL model 76510LV, Japan).

### Chlorophyll, photosynthesis measurement and leaf water potential

All the photosynthetic parameters were executed quantitatively. In the intact leaves the Soil Plant Analysis Development (SPAD) chlorophyll content was measured by using Minolta chlorophyll meter (SPAD-502; Konica Minolta Sensing Inc. Japan) whereas the transpiration rate (E), internal CO_2_ concentration (Ci), stomatal conductance (g_s_) and net photosynthesis rate (P_N_) were measured by Li-COR 6400 portable photosynthesis system (Li-COR, Lincoln, NE, USA). In the fresh leaf sample the LWP was measured by PSYPRO, water potential system, WESCOR, Inc, Logan, USA. All the physiological attributes was measured at 30 days after seeding.

### Measurement of fresh and dry matter of root and shoot and number of leaf per plant

Three plants of wheat from each pot for each inoculation were uprooted at 90 days after seeding and the length of root and shoot, dry matter of plant (root and shoot) and number of leaf per plant were measured by adopting the previous method^43^.

### Statistical Analysis

All experiments were performed at least three times independently with three to seven replications. Statistical analyses were performed using SPSS (Version 17) and Microsoft Office Excel 2007. Additionally, comparison of these means values was performed by conducting analysis of variance (ANOVA), followed by HSD (Turkey’s highest significant difference) assessment for the determination of significance (p ≤ 0.05).

## Data Availability

The dataset produced and analyzed during the present study are obtainable from the corresponding author on reasonable request.
